# The Truth Behind the Myth of Pomegranate Tree Root: Proofs on Anti-Nematode and Anti-Feeding Properties of Pelletierine-like Alkaloids

**DOI:** 10.3390/molecules31081254

**Published:** 2026-04-10

**Authors:** Sonia Bonacci, Pierpaolo Scarano, Giuseppe Iriti, Azucena Gonzáles-Coloma, María Fe Andrés, Carmine Guarino, Manuela Oliverio, Antonio Procopio

**Affiliations:** 1 Department of Health Sciences, University Magna Graecia, Viale Europa, Loc. Germaneto, 88100 Catanzaro, Italy; s.bonacci@unicz.it (S.B.); giuseppe.iriti@studenti.unicz.it (G.I.); procopio@unicz.it (A.P.); 2 Department of Sciences and Technologies, Università degli Studi del Sannio, 82100 Benevento, Italy; scarano@unisannio.it (P.S.); guarino@unisannio.it (C.G.); 3 Instituto de Ciencias Agrarias, Consejo Superrior de Investigaciones Cientificas, Serrano 115, 28006 Madrid, Spain; azu@ica.csic.es (A.G.-C.); mafay@ica.csic.es (M.F.A.)

**Keywords:** pelletierine, *Punica granatum* L., *Myzus persicae*, *Rhopalosiphum padi*, *Meloidogyne javanica*, nematicidal activity, antifeedant activity

## Abstract

Today, interest in natural remedies for biocontrol of crop pests is paramount. *Punica granatum* L. (pomegranate) is studied worldwide to obtain interesting bioactive compounds. Its anti-parasitic activity is associated with the presence of alkaloids in its roots. In this work, we explored the possibility of obtaining from *P. granatum* roots pelletierine-like alkaloids, which were extracted, characterized, isolated and used for the biocontrol of pests such as *Spodoptera littoralis*, *Myzus persicae*, *Rhopalosiphum padi* and *Meloidogyne javanica*. Two different extracts were obtained, characterised and quantified by GC-MS and LC-ESI-HRMS. In vitro assays of nematicidal activity were performed comparing the extracts with isopelletierine and pseudopelletierine as pure molecules. The results of these assays showed a difference in activity between iso- and pseudopelletierine, especially in terms of the nematocidal effect against *M. javanica* with isopelletierine being more active than pseudopelletierine. This leads us to conclude that only extracts from *P. granatum* roots with a high concentration of isopelletierine alkaloid can be used in effective pest control products.

## 1. Introduction

Today, pesticide overuse against pest infections of horticultural and fruit crops entails serious indirect environmental and economic costs, such as severe adverse effects on human health, poisoning of animals, contamination of agricultural products, progressive resistance of pests to pesticides, poisoning of pollinating insects and subsequent reduction of pollination, and contamination of ground and surface water [1,2]. Therefore, research is heading toward finding new plant-derived compounds to be used in safeguarding horticultural and fruit crops [3,4]. Often these compounds, have inherent in them, an effect called phytotherapeutic, which in this case becomes specifically related to pest management use [5,6]. Plants already do this work for their defense, making them potentially useful on a large scale is up to us. Therefore, these compounds have a well-defined nature and often act synergistically and not as a pure principle (although the action of the pure compound could potentially be more incisive against predatory insects or pests) [7,8].

*Punica granatum* L. was used in various regions in folkloric medicine as a treatment for many diseases, such as repellent against medically important mosquito vectors, parasitic worm infections, and protozoan parasite infections [9,10].

The antiparasitic activity of pomegranate against nematode gastrointestinal infestation of ruminants [11,12,13,14] as well as against horticultural crop nematodes [15,16,17,18,19], is well known by the literature. This activity seems to be due, among several biomolecules contained in *P. granatum* extracts, to alkaloids present in the bark of the root of *P. granatum* [20,21,22,23].

**Figure 1:**
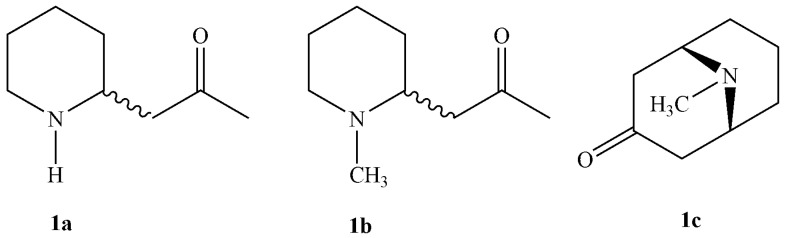
Chemical structures of isopelletierine (**1a**), *N*-methylpelletierine (**1b**) and pseudopelletierine (**1c**).

The most representative alkaloid in *P. granatum*. is pelletierine, which is a small molecule with an intriguing history. In the 1800s, Charles Joseph Tanret first extracted three compounds from pomegranate tree roots. Such compounds were named, in honour of botanist Pierre J. Pellier, as (±) pelletierine or isopelletierine (**1a**), *N*-methylpelletierine (**1b**), and pseudopelletierine (**1c**) (Figure 1).

Tanret described pelletierine as optically active, in contrast to isopelletierine. Later, biosynthetically derived (-)-pelletierine was isolated, but it underwent partial or complete racemization and, after salt formation with acids, crystallized as a racemic mixture. As subsequently shown, racemization of (-)-pelletierine is base-catalyzed. Therefore, the extent of conversion of (-)-pelletierine into isopelletierine depends on exposure to bases during the isolation process [24].

About 20 years passed before the structures of (-)-pelletierine and isopelletierine were clarified. Their exact structures were first elucidated when Jakob Meisenheimer, in 1921, reported the first synthesis of racemic (±)-pelletierine [25], thereby enabling its use as a building block for the synthesis of other molecules [26,27,28,29].

One of the earliest studies on the antiparasitic activity of pomegranate was that of von Schroeder [30]. That report did not specify which pomegranate part was active, nor whether pelletierine was present in the tested extracts. Zhicen later proposed that pelletierine is likely the principal agent responsible for pomegranate’s vermifugal and taeniacidal effects [31]. More recently, El Sakka argued that pelletierine, because of its structural similarity to strychnine, can provoke an enhanced stimulant reflex that may escalate to tetanic contraction, and suggested activity against various tapeworms, ringworms and nematodes [32]. However, these accounts provide few robust experimental data confirming the activity of isolated pelletierine, and studies using hydroalcoholic root extracts often lack detailed chemical characterization of their alkaloid composition.

Thus, critically analyzing the reported literature data, the antiparasitic activity of pelletierine seems to be, at least, a controversial topic and a myth poor of scientific justifications. Due to the controversial nature of the literature, a deeper investigation on the pelletierine activity as nematostatic/nematicidal is needed.

The aim of this work was to clarify the anti-parasitic activity of pelletierine and/or of other pelletierine-like alkaloids eventually present in higher amounts in *P. granatum* extracts, both as isolated molecules and as components of a root extract.

Studies were conducted on helminths and insects that infest horticultural crops, in particular *Meloidogyne javanica*, *Spodoptera littoralis*, *Myzus persicae*, and *Rhopalosiphum padi*. Two pomegranate root extracts, one obtained by a classical alkaloid extraction (AE) and the other by hydroalcoholic extraction (HAE), were chemically characterized by GC–MS and LC–ESI–HRMS, and their nematostatic/nematicidal activities were compared with those of pure pelletierine-type alkaloids.

## 2. Results and Discussion

### 2.1. Chemical Characterization of Pomegranate Root Bark Extract

Before investigating the nematostatic/nematicidal activity of pelletierine-like alkaloids, we verified their presence in both AE and HAE root extracts. We hypothesized that, being small and hydrophobic, these alkaloids would be scarce in HAE but abundant in AE.

In order to well clarify the composition of the tested root extracts, GC-MS and LC-ESI-HRMS analysis of HAE and AE were performed.

**Figure 2:**
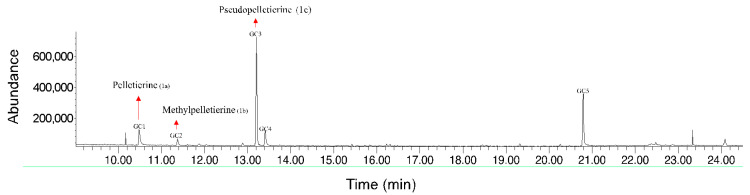
GC-MS chromatogram of pomegranate root AE analysis containing the alkaloids belonging to the pelletierine family: (**1a**) isopelletierine; (**1b**) methylpelletierine; (**1c**) pseudopelletierine.

GC-MS analysis of AE revealed compounds of the pelletierine family: pelletierine (**1a**), methylpelletierine (**1b**), and pseudopelletierine (**1c**) (Figure 2).

Isopelletierine (**1a**), methylpelletierine (**1b**) and pseudopelletierine (**1c**) were quantified as a relative percentage of the total peak area. Table 1 reports the identification and relative percentage of the most representative compounds in AE (see Supporting Information for the detailed EI/MS spectra of the identified peaks, Figures S2–S6).

As expected, the AE profile was relatively simple, reflecting the method’s selectivity for alkaloids (Figure 2). Pelletierine-family alkaloids comprised over half of the detected compounds (56.23%, Table 1), with pseudopelletierine (**1c**) the most abundant (44.37%, Table 1). Isopelletierine (**1a**) was a minor component (8.77%, Table 1), consistent with its metabolic conversion to other derivatives. Given its low abundance and the basic extraction conditions, we did not pursue stereochemical analysis of pelletierine and assumed it was fully racemized to isopelletierine.

LC-ESI–HRMS analysis of AE, performed to detect highly volatile phenols, did not reveal other compound classes at significant levels.

**Figure 3:**
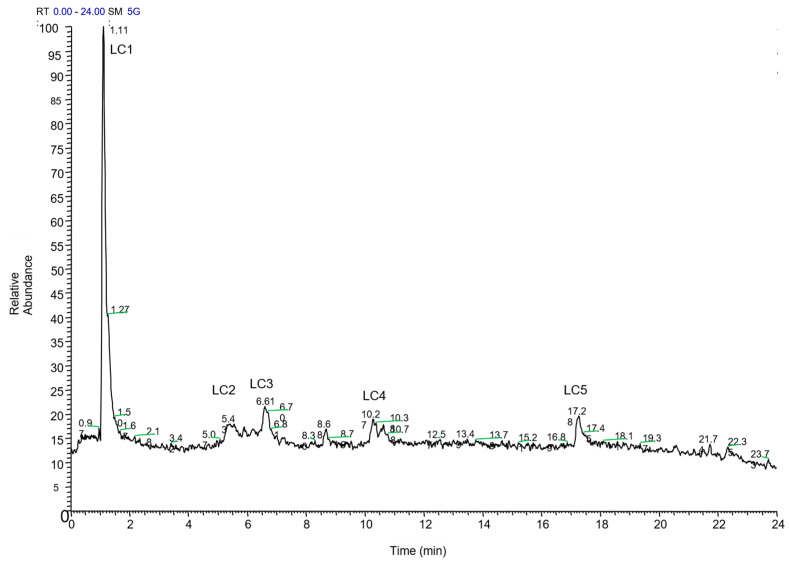
Full MS chromatogram of LC-ESI-HRMS analysis of hydroalcoholic (HAE) extract of pomegranate root: (LC**1**) 3-(4-hydroxyphenyl) lactic acid; (LC**2**) punicalagin; (LC**3**) gallocatechin; (LC**4**) epicatechin; (LC**5**) quercetin.

GC-MS analysis of HAE was carried out with a six-minute solvent delay to enhance peak detection. Unlike AE, HAE showed no alkaloids or other volatile compounds (see Supporting Information, Figure S7). To assess non-volatile constituents, we performed LC-ESI–HRMS; the full-MS scan is shown in Figure 3 (HRMS spectra of all identified compounds in Supporting Information, Figures S8–S12), confirming the presence of non-volatile phenols in HAE.

Table 2 summarizes the main detected fragments useful to confirm detected phenols in HAE, quantified as a relative percentage of the total peak area. Among the identified compounds, compound LC2 (Figure 2, Table 2) with a double charge molecular ion at *m*/*z* 541 was identified as punicalagin. Its mass spectrum showed typical daughter fragments at *m*/*z* 601 and 301 as previously described by Sentandreu et al. [33]. Moreover, as pointed out by Elshamy et al., [34] gallocatechin (compound LC3, Figure 2, Table 2) with a negative molecular ion at *m*/*z* 305 and epicatechin (compound LC4) with *m*/*z* 289 were identified and confirmed by characteristic fragments reported in Table 2. Quercetin was identified by comparison with a commercial standard.

After characterization, the main alkaloid from AE, pseudopelletierine (**1c**), was purified to allow comparison of its nematicidal and anti-feeding effects with those of the extracts. Pseudopelletierine was isolated by flash liquid chromatography, and its purity was verified by GC–MS (see Supporting Information, Figure S13). Isopelletierine (**1a**), which was present in AE at lower abundance, was synthesized and purified according to the literature method [35] (see Supporting Information, Scheme S1 and Figure S14) to provide sufficient material for comparison. Methylpelletierine (**1b**), being a minor component, was not further investigated; the literature focuses on pelletierine and does not distinguish activities of isopelletierine (**1a**) and pseudopelletierine (**1c**), nor reports data for methylpelletierine as a pure compound.

### 2.2. Antifeedant Effect

Pure compounds and pomegranate extracts were subjected to preliminary antifeedant bioassays against three insect species (*S. littoralis*, *R. padi*, and *M. persicae*). For samples exhibiting %SI (or %FI) ≥ 60% at 50 µg·cm^−2^, dose–response curves were determined and EC_5_0 values calculated. The results are summarized in Table 3.

No effect was observed on *S. littoralis* (Table 3). In contrast, isopelletierine (50.0 µg·cm^−2^) showed notable activity against *R. padi* and *M. persicae*, with %SI values of 63.32 ± 6.38 and 54.57 ± 7.71, respectively (not further investigated here). Dose-response analysis indicated species-specific sensitivity: at the highest tested dose (50 µg·cm^−2^) the EC_50_ values were 0.864 mg·cm^−2^ for *R. padi* and 1.441 mg·cm^−2^ for *M. persicae*, while lower concentrations were inactive [36]. These preliminary results suggest potential for control of these pests, particularly *R. padi* and *M. persicae*, either by applying the pure compound at moderately higher doses (e.g., 75–100 µg·cm^−2^) or in combination with other alkaloids, pending targeted studies [37,38,39]. Further investigations into the mechanism of action are required to explain the selectivity for *R. padi* and *M. persicae* versus *S. littoralis*.

Concerning pure pseudopelletierine and pomegranate extracts, both AE and HAE, they were less active against all the tested pests than isopelletierine (Table 3). This result evidenced for the first time that isopelletierine and pseudopelletierine, even belonging to the same family, have different efficacy as antifeedant agents. The AE, being composed of pseudopelletierine as the main component, showed moderate activity, in between the two pure molecules. In the cases of *R. padi* and *M. persicae*, we can observe a slightly greater efficacy, probably due to the aforementioned synergistic action; this, however, is not evident for *S. littoralis*.

The potential of *P. granatum* extracts against certain insects has been reported, for example, efficacy was observed against *Rhynchophorus ferrugineus* [40] for the extracts but not for the pure compound. However, our results indicate that extract composition is critical: differences in the relative proportions of isopelletierine and pseudopelletierine may lead to markedly different bioactivity outcomes.

### 2.3. In Vitro Nematicidal Effect

The in vitro nematicidal effects against *M. javanica* of the tested samples are shown in Table 4. The HAE, AE, isopelletierine (**1a**) and pseudopelletierine (**1c**) samples were tested.

The results obtained for all tested samples confirmed the behavior already observed in the antifeedant test as regard to isopelletierine, which was the most active molecules with a LC_50_ of 0.163 mg·mL^−1^ and a LC_90_ of 0.389 mg·mL^−1^, comparable to the positive control. Surprisingly, despite the low amount of isopelletierine in its composition (quite 10% *w*/*w*), the AE resulted also active against *M. javanica* with a LC_50_ of 0.256 mg·mL^−1^ and a LC_90_ of 0.566 mg·mL^−1^, the 50% less respect to the pure molecule. In this case the high sensibility of *M. javanica* towards isopelletierine was more significant than the inefficacy of pseudopelletierine, thus don’t compromising the activity of the whole phytocomplex.

The HAE of *P. granatum*, it was ineffective even in this case.

As a perennial crop, *P. granatum* remains in the same field for many years and can therefore act as a persistent host for several species of root-knot nematodes belonging to the genus *M. javanica*. The long-term presence of the host favors the gradual build-up of nematode populations in the soil, ultimately leading to high infestation levels that negatively affect root development and reduce the efficiency of water and nutrient uptake. Infection typically results in the formation of characteristic root galls, which impair root functionality and progressively weaken the plant. Moreover, the damage caused by nematode feeding creates entry points for soilborne pathogens, including opportunistic fungi and bacteria, thereby increasing the likelihood of secondary infections [42,43]. These combined stresses can intensify disease severity, accelerate plant decline, and in severe cases lead to plant death. Therefore, root-knot nematode infestations represent a major constraint for the productivity, health, and longevity of pomegranate orchards, particularly under intensive cultivation systems. In this context, the development and adoption of sustainable management strategies aimed at reducing nematode pressure and improving plant resilience are of considerable importance.

Previous non-specific studies [15] evaluated *P. granatum* extracts against *M. javanica*. In this work, root bark extracts were investigated specifically, and the role of alkaloids was examined in detail. By comparing extracts with the corresponding pure compounds, pelletierine-family alkaloids were identified as the targeted agents against this pest: pure isopelletierine showed activity against *M. javanica*, whereas pseudopelletierine did not. The predominance of an ineffective alkaloid in the AE reduced the activity of the whole phytocomplex (EC values: 0.163 for pseudopelletierine versus 0.256 for isopelletierine; pseudopelletierine was inactive). These data indicate that pure isopelletierine, rather than its metabolic derivatives or tannin-derived phenols, is the principal nematostatic/nematicidal constituent in pomegranate roots. Further studies on the natural stereochemistry are required to assess the influence of enantiomeric purity on activity. Finally, the nematicidal effects reported for high-concentration aqueous or hydroalcoholic extracts of pomegranate root bark and other fruit parts [14,15,16,17,18,19,20,21,22] are likely attributable to other compound classes, such as phenols [44].

This certainly opens new avenues in obtaining and using such selected compounds, also obtained from waste matrices or endemic plants, in the fight against pest infestations [45,46].

## 3. Materials and Methods

*General*. All chemicals were obtained from Merck (KGaA, Darmstadt, Germany) and used as received. Dried pomegranate root bark and the hydroalcoholic extract of pomegranate tree root were provided by Phytocal s.r.l. (Rende, CS, Italy).

All reactions were monitored by TLC on silica Merck 60 F254 precoated aluminum plates (KGaA, Darmstadt, Germany).

GC-MS analyses were performed by a 6890N (Agilent Technologies, Santa Clara, CA, USA) equipped with a Varian VF-5m capillary column (30 m × 0.25 mm × 0.25 μm), coupled to a single quadrupole mass selective detector 5973 Network (Agilent Technologies).

Liquid chromatography was performed on a Thermo Scientific (Rodano, MI, Italy) Dionex Ultimate 3000 RS connected to a Thermo Scientific Hypersil Gold C18 column (50 × 2.1 mm, 1.9 μm particle size). Ultra grade LC/MS solvents and ultrapure water, obtained from a Milli-Q Integral 5 system (Millipore, Merck KGaA Darmstadt, Germany), were used as eluents. High Resolution Mass Spectrometry (HRMS) was performed on a Thermo Scientific Q-ExactiveTM (Rodano, MI, Italy) mass spectrometer.

*Alkaloids Extract (AE) from the pomegranate root bark.* The selective extraction of alkaloids presents in *P. granatum* root bark was performed as reported by Sicker et al. 2019 [24]. The shredded root bark of the pomegranate tree (53 g) was pulverized to a coarse powder in a kitchen mill. CaO (20.0 g), NaOH (1.0 g) and H_2_O (145 mL) were mixed to obtain a suspension of low viscosity, to which was then added the root bark, resulting in a paste-like mixture ochre to red-brown in color. The mixture was stirred overnight in an ice bath, to make it more homogenous and less viscous. The suspension was diluted with water (285 mL), and the solids were removed by filtration under suction. The filtration was repeated five times to obtain a clear filtrate, which was then extracted four times with chloroform (4 × 200 mL). The colourless organic phases was dried over MgSO_4_ and filtered. The solvent was removed to dryness under reduced pressure, to obtain a yellowish oil (85.5 mg). The oil was dissolved in chloroform (10 mL) and extracted twice with 20% H_2_SO_4_ (2 × 5 mL). The sulphuric acid phase was cooled in an ice bath and aq. NaOH (4.6 M) was added in small drops to attain a pH of 11. The resulting precipitate of Na_2_SO_4_ was removed by filtration under suction, and the aqueous solution was extracted three times with dichloromethane (6 × 20 mL). The ether phase was dried over MgSO_4_ and filtered, and the solvent was removed to dryness under reduced pressure. The yellowish oil (31 mg) obtained, according to TLC, contains pseudopelletierine as major component (DCM/MeOH 6:4, 1% of triethylamine) [24]. The extract was then concentrated under vacuum and analyzed by GC-MS and LC-ESI-HRMS. Isopelletierine (**1a**), methylpelletierine (**1b**) and pseudopelletierine (**1c**) were quantified as a relative percentage of the total peak area (see Supporting Information, Figures S1–S6). Due to the basic conditions of this extraction, racemization of pelletierine was assumed, but no further analysis to verify it were performed as isopelletierine was the minor component of the mixture. A portion of the extract was used to purify the major component pseudopelletierine (**1c**) by flash liquid chromatography on silica gel (Ch_2_Cl_2_/MeOH 8:2 *v*/*v* + 2% of triethylamine as eluent). Isolated pseudopelletierine was characterized by GC/MS while ^1^H-NMR was compared with the data reported in the literature [24].

*Hydroalcoholic Extraction (HAE) from pomegranate tree root.* The extraction was carried out by Phytocal s.r.l. as follow: fresh root was washed, shredded, and macerated in a hydroalcoholic solution (EtOH/H_2_O 50:50, *v*/*v*) for 21 days at room temperature. Consequently, the extract was concentrated under vacuum and analyzed by GC-MS and LC-ESI-HRMS (see Supporting Information, Figures S7–S12).

*GC-MS analysis of root extracts.* GC-MS analyses were performed using the following setup: helium was used as the carrier gas at a flow of 0.8 mL·min^−1^; the temperature of the injector and transfer-line was 250 °C. The split mode was used for injection with a split ratio of 40:1. The oven temperature program was as follows: initial temperature of 35 °C, held for 1 min, increased from 35 °C to 250 °C at 10 °C·min^−1^, held for 10 min, finally raised to 290 °C at 5 °C·min^−1^, and held for 5 min. Volume injected was 1 μL. The operating conditions of the MS were the following: ionization potential 70 eV, source temperature 250 °C, solvent delay 16.5 min, mass range 50–500 *m*/*z*. The products were identified by comparison with the NIST database.

*LC-ESI-HRMS analysis of root extracts.* Liquid chromatography was performed on a Hypersil Gold C18 column (50 × 2.1 mm, 1.9 μm particle size). The temperature of the column and the autosampler were maintained at 24 °C and 4 °C, respectively. The column was equilibrated in 98% solvent A (water with 0.1% (*v*/*v*) formic acid) and in 2% solvent B (methanol). The elution flow rate was 300 μL·min^−1^. Analysis were performed according to the following solvent gradient: equilibration for 2 min with 2% solvent B, then solvent B was increased from 2 to 23% in 6 min, isocratic for 5 min, then increased from 23% to 50% in 7 min and from 50% to 98% in 5 min, isocratic for 6 min and then decreased until the initial conditions (2% B) in 6 min and isocratic for 3 min. High Resolution Mass Spectrometry (HRMS) was performed working with an electrospray source (spray voltage 3.0 kV, sheath gas: 20, arbitrary units, Auxiliary gas: 8, probe heater temperature: 280 °C; capillary temperature: 320 °C; S-Lens RF Level: 50), with both negative and positive polarities, at 70,000 resolving power (defined as FWHM at *m*/*z* 200), IT 100 ms, and ACG target = 3 × 106, by full scan analysis with a mass range from 50 to 750 *m*/*z*. Nitrogen high-purity gas was used as both sheath gas and auxiliary gas. The instrument was daily calibrated by Thermo ESI calibration solution.

*Synthesis of isopelletierine.* The synthesis of isopelletierine (**1a**) was carried out as reported by Quick et al. [35] (See Supporting Information, Scheme S1). Briefly, a rapidly stirred suspension of *N*-chlorosuccinimide (4.72 g, 6 mmol) in ether (100 mL) was added piperidine (2 mL, 20 mmol). After one hour, the mixture was filtered, and the filtrate was washed twice with water (2 × 100 mL), once with aqueous sodium chloride (100 mL), and finally dried with dry MgSO_4_. The ether solution of *N*-chloropiperidine was concentrated (15 mL) and added drop-wise over a period of 1 h to a stirred solution of potassium hydroxide (1.12 g, 20 mmol) in absolute ethanol (10 mL), kept at 5 °C with an ice bath. The mixture was stirred at room temperature for 14 h (shorter times can be used) and then filtered. The filtrate (25 mL), containing piperidine, was added to an aqueous solution of sodium acetoacetate, prepared by heating for 4 h at 50 °Can aqueous solution of ethyl acetoacetate (2.6 g, 20 mmol) and sodium hydroxide (1.2 g, 30 mmol) in water (40 mL). This mixture was refluxed for 4 h. After cooling, the ether and most of the ethanol were removed by concentration under vacuum. The resulting aqueous solution was extracted three times with dichloromethane (200 mL) and finally dried on dry MgSO_4_. The crude (1.247 g) was purified by flash chromatography (eluent DCM/MeOH 6:4 *v*/*v*, 1% of triethylamine). Isopelletierine was characterized by GC-MS (See Supporting Information, Figures S13 and S14), while ^1^H-NMR was compared with the data reported in the literature [35].

*Insect and Plant Material.* Colonies of *Spodoptera littoralis* (Lepidoptera, Noctuidae) were reared on an artificial diet as described previously [47]. Colonies of *Myzus persicae* (Homoptera, Aphididae) and *Rhopalosiphum padi* (Hemiptera, Aphididae) were maintained on bell pepper (*Capsicum annuum* L. var. Califronia Wonder) and barley (*Hordeum vulgare* L.) plants, respectively. The insect colonies were raised and maintained at the ICA-CSIC (Instituto de Ciencias Agrarias—Consejo Superior de Investigaciones Científicas, in Madrid).

Host plants were grown from seeds in pots containing commercial substrate and periodically infested with aphids: *C. annuum* plants at the 4-leaf stage and *H. vulgare* plants when reaching approximately 10 cm in height. Both insect colonies and host plants were maintained in a growth chamber at 22.00 ± 1.00 °C and relative humidity (RH) > 70%, with a photoperiod of 16:8 h (L:D).

*Antifeedant Activity.* The bioassays were conducted as described for Navarro-Rocha et al. [48] Biological tests were conducted with adult specimens of *M. persicae* (20 replicates with 10 or more insects each) and *R. padi* (20 replicates with 10 or more insects each) aged 24 to 48 h, with larvae at least in the sixth stage > 24 h after molting for *S. littoralis* (out of 10 replicates with 2 insects each). Sections of *C. annuum* and *H. vulgare* leaves (1.0 cm^2^) were prepared in the tests: the upper surface of the sections, discs or leaf fragments (See Supporting Information, Figures S15–S17), were treated with 10 µL of the extract sample or the pure substance or control. Each sample (Pseudopelletierine, Isopelletierine, HAE or AE extracts) was tested with a dose of 10 µg/μL (50 µg·cm^−2^). A total of 20 cubic, ventilated plastic boxes (2 × 2 cm^2^) with 10 aphids, 6 petri dishes (10 cm^2^) with 2 *S. littoralis* larvae were tested. The experiment on *S. littoralis* is carried out by placing the larvae in petri dishes with the leaf sections on which the control and sample were placed, in opposite positions, where the larvae are allowed to feed: consumption is allowed up to 75% consumption of either control or sample discs. For aphids, on the other hand, they are placed in the experiment boxes for 24 h, at the end of which counting is done on the leaf sections with sample and control. Each experiment was repeated 3 times. Feeding inhibition (FI) and settling inhibition (SI) of aphids was calculated: it was by measuring disc surface consumption for *S. littoralis* and calculated the number of aphids settled on leaf section surfaces for *M. persicae* and *R. padi* (photo files were digitized with https://imagej.net/ij/ accessed on 29 march 2025) [49].

Feeding/decantation inhibition (%FI or %SI) was calculated as %FI or %SI = [1 − (T/C) × 100], where T and C represent feeding/decantation on treated and control leaf discs. Extracts and compounds with a %FI or %SI > 60% were further tested in additional dose-response experiments: serial 1:2 dilutions were made until the recorded activity was in the range of 100.00–50.01% dietary inhibition/decantation, for a minimum of 3 doses; based on the results obtained, their effective dose, EC_50_ (dose to achieve 50% stabilization reduction), was calculated using linear regression analysis (% FI/SI on logarithmic dose) [36] (STATGRAPHICS Centurion XVI, version 16.1.02, The Plains, VA, USA).

Based on the results, the predicted antifeedant effect ((%FI or %SI)/cm^2^) was calculated for each compound at the initial concentration of 50 µg/cm^−2^ as follows:

Predicted %FI (or %SI) (50 µg/cm^2^) = [(%Compound/100) × 50]/EC_50_

*Nematicidal Activity and nematicide Bioassay.* The nematode population (*Melodogyne javanica*) used for the experiment was maintained and grown on plastic pots of tomato plants (*Solanum lycopersicum* var. Marmande) in a growth/cultivation chamber (with temperature and relative humidity, of 25.00 ± 1.00 °C and 70%, respectively).

Bioassays were prepared by manually recovering *M. javanica* egg masses from Marmande’s root galls, then soaking them for 24 h in sterile water at 25.00 ± 1.00 °C (See Supporting Information Figure S18).

The solution containing the hatched second stage juveniles (J2) was adjusted to a final concentration of 100 J2/100 μL in distilled water [50,51,52,53].

A 96-well plate with a U-bottom was prepared (SARSTEDT AG & Co. KG, Nümbrecht, Germany) (See Supporting Information, Figure S4): 100 µL of sample (Pseudopelletierinee, Isopelletierinee, HAE or AE extracts) and 100 µL of nematode suspension (containing approximately 100 J2) were placed in each well. A suspension of nematodes in distilled water (100 J2/100 µL) was used as a negative control, a thymol solution (LC_50_ = 0.143 mg·mL^−1^) was used as a positive control. Four replicates were carried out for each treatment. The plates thus prepared were sealed and incubated in the dark using the same conditions used for the incubation of egg masses described by Moo-Koh et al. [52].

The isopelletierine, HAE and AE samples, with a maximum concentration of 2 mg·mL^−1^, were dissolved in a 0.5% EtOH hydroalcoholic solution. The final concentration obtained was 1 mg·mL^−1^ (100 µL of sample and 100 µL of nematode suspension).

The dead J2s were counted (Monitoring of the plate at 72 h to identify dead J2s was carried out using an optical microscope and by stimulating the specimens with the tip of a needle) expressing the result as percentage mortality (M%) [51]: the nematicidal activity was presented as the correct percentage of J2 mortality, according to the formula by Schneider-Orelli [53]:

% efficiency = [(b − k)/(100 − k)] × 100

where b represents the % of individuals who died in the treatment and k represent the % of individuals who died in the control.

Six serial concentrations (1.0, 0.50, 0.25, 0.13, 0.063, and 0.031 mg·mL^−1^) (0.50 mg/mL) of those treatment showing 100% of mortality were tested to obtain an effective lethal concentration. The LC_50_ and LC_90_ values were determined using a Probit analysis (STATGRAPHICS Centurion XVI, version 16.1.02, The Plains, VA, USA).

## 4. Conclusions and Future Perspectives

In conclusion, the activity of pure isopelletierine (**1a**) as both antifeedant agent against *R. padi* and *M. persicae* and as nematicidal agent against *M. javanica* was demonstrated. Further investigations are needed to clarify the mechanisms of such actions, especially to justify the selectivity respect the pests and the inactivity against *S. littoralis.* The identification of the main components in the AE and HAE extract of pomegranate root bark clarified that nor isopelletierine (**1a**) or other alkaloids belonging to the pelletierine-like family are present in HAE, while pseudopelletierine (**1c**), a pelletierine metabolite, is the major component of AE. The comparison between the activity of pure pseudopelletierine (**1c**) and isopelletierine (**1a**) evidenced that pseudopelletierine (**1c**) has only a moderate activity as antifeedant agent against *R. padi* and *M. persicae*, while it is completely ineffective as nematicidal agent against *M. javanica.* This negatively influenced the activity of the AE, especially concerning to the antifeedant effect against *R. padi* and *M. persicae* (although with a slightly higher result than the pure molecule), while an activity as nematicidal agent against *M. javanica* is preserved. Such results allowed to infer that the previously reported data on the pesticide activity of *P. granatum* L. rootbark extracts, mainly hydroalcoholic, must be due to other bioactive molecules different from pelletierine-like alkaloids. This leads us to conclude that only extracts of *P. granatum* L. roots with a high concentration of isopelletierine alkaloid can be used in effective pest control products.

On the other hand, the use of pure isopelletierine for controlling parasitic nematodes in agriculture could represent a significant agronomic breakthrough. From an agronomic perspective, the introduction of natural compounds such as isopelletierine for the management of phytoparasites [54] addresses the growing demand for sustainable solutions that reduce reliance on synthetic chemical nematicides like fenamiphos or oxamyl, which are often associated with phytotoxicity and environmental risks. Commercially, the use of isopelletierine in granular or liquid formulations suitable for soil application could open new market opportunities in the biopesticide sector, particularly appealing to organic and integrated farming systems. Production costs, estimated based on extraction from pomegranate roots, range between €60 and €100 per kilogram of pure active ingredient, with application costs (at effective doses of 1–2 kg/ha) around €120–200 per hectare, competitive with conventional nematicides.

From an environmental standpoint, pelletierine presents a lower impact than persistent chemicals: it is biodegradable, non-bio accumulative, and has a relatively short half-life in soil (approximately 10–14 days), reducing the risk of water contamination. However, the alkaloid shows moderate toxicity to other organisms: the oral LD_50_ in rodents is about 70 mg/kg, indicating a non-negligible acute toxicity if not handled according to proper safety protocols [55]. In comparison, the LD_50_ in target parasites such as *Meloidogyne incognita* is around 5–10 mg/kg, suggesting a favorable selectivity margin, (this is a hypothetical estimate based on data available for other plant compounds, including alkaloids) [44,56]. This relatively low LD_50_ in nematodes, compared to that in mammals and beneficial insects, represents a theoretical advantage for targeted application, though it also calls for caution regarding human exposure and field dosing.

In summary, pelletierine may represent a promising alternative for sustainable pest management, potentially providing both agronomic and environmental benefits. Nevertheless, before its large-scale adoption, further research is needed to address key aspects such as formulation optimization, environmental toxicology, and compliance with regulatory frameworks. One particularly promising research direction involves the selection and promotion of pomegranate cultivars characterized by a high pelletierine content. Such an approach could enhance the plant’s natural capacity to counter soil-borne pests. Future studies should therefore investigate the role of pelletierine in plant defence mechanisms and evaluate the feasibility of integrating varietal selection with appropriate agronomic practices and integrated pest management strategies. Ultimately, this integrated approach could support the development of more resilient and sustainable production systems for pomegranate cultivation and potentially for other crops as well.

## Figures and Tables

**Table 1:** Identification of the main peaks in pomegranate root AE by GC-MS.

Peak	Retention Time (min)	Identification	Molecular Formula	Relative Percentage (%)
GC1	10.49	Isopelletierine (**1a**)	C_8_H_15_NO	8.77
GC2	11.38	Methylpelletierine (**1b**)	C_9_H_17_NO	3.09
GC3	13.21	Pseudopelletierine (**1c**)	C_9_H_15_NO	44.37
GC4	13.41	*trans*-benzylidenacetone	C_10_H_10_O	8.82
GC5	20.79	heptadeca-5,8-dione	C_17_H_32_O_2_	21.70
others	-	-	-	13.25

**Table 2:** Identified metabolites in pomegranate root HAE.

Peak	Retention Time (min)	Identification	Molecular Formula	[M-H]^−^/[M-H]^−2^ (*m*/*z*)	Main Detected Fragments (*m*/*z*)	Relative Percentage (%)
LC**1**	1.11	3-(4-hydroxyphenyl) lactic acid	C_9_H_10_O_4_	181.0769	163.0593, 101.0255, 89.0255, 71.0121	76.36
LC**2**	6.61	punicalagin	C_48_H_28_O_30_	1083.0586 541.0262	781.0529, 600.9899, 541.0262, 300.9901, 275.0198	7.83
LC**3**	8.68	hexahydroxyflavan isomer (gallocatechin)	C_15_H_14_O_7_	305.0702	225.1129, 96.9588	3.70
LC**4**	10.24	epicatechin	C_15_H_14_O_6_	288.9991	245.0090, 217.0137	4.63
LC**5**	17.28	quercetin	C_15_H_10_O_7_	300.9999	-	7.47

**Table 3:** Insect antifeedant (against *S. littoralis*, *R. padi* and *M. persicae*) effect of tested samples.

Treatments	Conc. (µg·cm^−2^)	*S. littoralis*	*R. padi*	*M. persicae*
		%FI ^1^	%SI ^1^	%SI ^1^
Isopelletierine (**1a**)	50.0	33.52 ± 11.55	63.32 ± 6.38 * 0.864 (0.354–2.112) ^2^	54.57 ± 7.71 * 1.441 (0.732–2.836) ^2^
	25.0	n.t. ^3^	43.06 ± 6.99	33.24 ± 6.69
	12.5	n.t. ^3^	n.t. ^3^	n.t. ^3^
	6.25	n.t. ^3^	n.t. ^3^	n.t. ^3^
Pseudopelletierine (**1c**)	50.0	41.59 ± 18.68	49.24 ± 7.92	38.71 ± 8.70
AE	50.0	33.44 ± 7.25	59.55 ± 8.69	47.20 ± 9.18
HAE	50.0	22.56 ± 15.90	48.35 ± 7.84	53.80 ± 9.42
Control:	-	0.00	89.01 ± 1.86	83.94 ± 4.07

^1^ Percent feeding (FI) or setting (SI) inhibition at a maximum dose of 100 µg·cm^−2^. Values are means of 10 or 20 replicates, respectively. ^2^ EC_50_ expressed in mg·cm^−2^ (95% lower-upper confidence limits, CL), was the concentration needed to produce 50% FI or SI. At least six concentrations/dilutions were used [36] ^3^ n.t. = not tested. Values with an asterisk (*) are significantly different according to Wilcoxon paired rank test (*p* < 0.05).

**Table 4:** Nematicidal effects of isopelletierine and extracts from *P. granatum* root bark against second-stage juveniles (J2) of *M. javanica* after 72 h of exposure.

Treatments	J2 Mortality (M%)			
	Conc. % (*w*/*v*) ^1^	Eff. (%) ^2^	LC_50_ (95% CL) ^3^	LC_90_ (95% CL)
Isopelletierine (**1a**)	1.0	100	0.163 (0.123–0.217)	0.389 (0.293–0.517)
	0.50	100		
	0.25	100		
	0.13	5.98 ± 1.27		
	0.063	3.65 ± 1.38		
	0.032	1.27 ± 0.15		
Pseudopelletierine (**1c**)	1.0	5.96 ± 1.42	n.t. ^4^	n.t. ^4^
AE	1.0	100	0.256 (0.192–0.340)	0.566 (0.425–0.753)
	0.50	100		
	0.25	100		
	0.13	48.75 ± 6.47		
	0.063	12.13 ± 2.48		
	0.032	1.27 ± 0.15		
HAE	1.0	6.43 ± 1.93	n.t. ^4^	n.t. ^4^
Positive control: thymol	1.0 ^5^	100	0.143 (0.137–0.148)	0.195 (0.188–0.204)

^1^ Tested concentration: undiluted sample at 1.0 mg·mL^−1^. ^2^ Percent mortality at a dose of 1.0 mg·mL^−1^; values (%) are means ± standard error of four replicates. ^3^ At least six concentrations/dilutions were used, at 72 h, to obtain LC_50_ and LC_90_, expressed as mg·mL^−1^; CL denotes confidence limit. ^4^ n.t. = Not tested ^5^ As reported for Nasiou E. and Giannakou O., 2023 [41].

## Data Availability

Data supporting reported results can be provided by the authors upon request.

## Supplementary Materials

The following supporting information can be downloaded at: https://www.mdpi.com/article/10.3390/molecules31081254/s1, Figure S1. Full chromatogram, extract chromatogram and integrated peaks table; Figure S2. MS spectrum of GC1-Isopelletierine (**1a**); Figure S3. MS spectrum of GC2-Methylpelletierine (**1b**); Figure S4. MS spectrum of GC3-Pseudopelletierine (**1c**); Figure S5. MS spectrum of GC4; Figure S6. MS spectrum of GC5; Figure S7. GC-MS analysis of hydroalcoholic extract of pomegranate root; Figure S8. MS spectrum of LC1; Figure S9. MS spectrum of LC2; Figure S10. MS spectrum of LC3; Figure S11. MS spectrum of LC4; Figure S12. MS spectrum of LC5; Figure S13. GC-MS chromatogram of isolated pseudopelletierine (**1a**); Scheme S1. Synthesis of isopelletierine (**1a**); Figure S14. GC-MS chromatogram of synthesized isopelletierine (**1a**); Figure S15. Preparation of anti-food experiment for *Spodoptera littoralis*; Figure S16. Preparation of anti-stick experiment for *Myzus persicae*; Figure S17. Preparation of anti-stick experiment for *Rhopalosiphum padi*; Figure S18. Experiments performed in 96-well plastic plates (U-bottom) with 4 replicates per treatment plus control on *M. javanica.*

## Abbreviations

The following abbreviations are used in this manuscript:

GC-MS	Gas Chromatography Mass Spectrometry
LC-ESI-HRMS	Liquid Chromatography Electron Spray Ionization High resolution mass Spectrometry
AE	Alkaloids Extract
HAE	Hydro-Alcoholic Extract
EI-MS	Electronic Ionization Mass spectrometry
EC_50_	Effective Concentration (50% inhibition)
LC_50_	Lethal Concentration (50% mortality)
LC_90_	Lethal Concentration (90% mortality)
FI	Feeding Inhibition
SI	Settling Inhibition
LD_50_	Lethal Dose (50% mortality)
