# Supplementary material for: The Truth Behind the Myth of Pomegranate Tree Root: Proofs on Anti-Nematode and Anti-Feeding Properties of Pelletierine-like Alkaloids
Source: Molecules. 2026 Apr 10;31(8):1254. doi: 10.3390/molecules31081254 (PMC13118509; doi:10.3390/molecules31081254)
Supplement: Supplementary file 1 [file molecules-31-01254-s001.zip › molecules-4210828-supplementary.pdf]

Supporting Information

The truth behind the myth of pomegranate tree root: proofs on anti-nematode and anti-feeding properties of pelletierine-like alkaloids.

Sonia Bonacci,[a] Pierpaolo Scarano,[b] Giuseppe Iriti,[a] Azucena Gonzáles-Coloma,[c] María Fe Andrés,[c] Carmine Guarino,[b] Manuela Oliverio,*[a] Antonio Procopio.[a]

[a]Department of Health Sciences, University Magna Graecia, Viale Europa, Loc. Germaneto, 88100, Catanzaro, Italy. Email: m.oliverio@unicz.it Phone number: +39.0961.3694121

[b]Department of Sciences and Technologies, Università degli Studi del Sannio, 82100 Benevento, Italy.

[c] Instituto de Ciencias Agrarias, Consejo Superrior de Investigaciones Cientificas, Serrano 115, 28006, Madrid, Spain.

Figure S1. Full chromatogram, extract chromatogram and integrated peaks table pag 2

Figure S2. MS spectrum of GC1- Isopelletierine (1a). pag 2

Figure S3. MS spectrum of GC2- Methylpelletierine (1b) pag 3

Figure S4. MS spectrum of GC3- Pseudopelletierine (1c). pag 3

Figure S5. MS spectrum of GC4- pag 4

Figure S6. MS spectrum of GC5 pag 4

Figure S7. GC-MS analysis of hydroalcoholic extract of pomegranate root pag 5

Figure S8. MS spectrum of LC1 pag 6

Figure S9. MS spectrum of LC2 pag 6

Figure S10. MS spectrum of LC3 pag 7

Figure S11. MS spectrum of LC4 pag 7

Figure S12. MS spectrum of LC5 pag 8

Figure S13. GC-MS chromatogram of isolated pseudopelletierine (1a) pag 9

Scheme S1. Synthesis of isopelletierine (1a). pag 10

Figure S14. GC-MS chromatogram of synthesized isopelletierine (1a) pag 10

Figure S15. Preparation of anti-food experiment for *Spodoptera littoralis* pag 11

Figure S16. Preparation of anti-stick experiment for *Myzus persicae* pag 11

Figure S17. Preparation of anti-stick experiment for *Rhopalosiphum padi*. pag 12

Figure S18. Experiments performed in 96-well plastic plates (U-bottom) with 4 replicates per treatment plus control on *M. javanica* pag 12

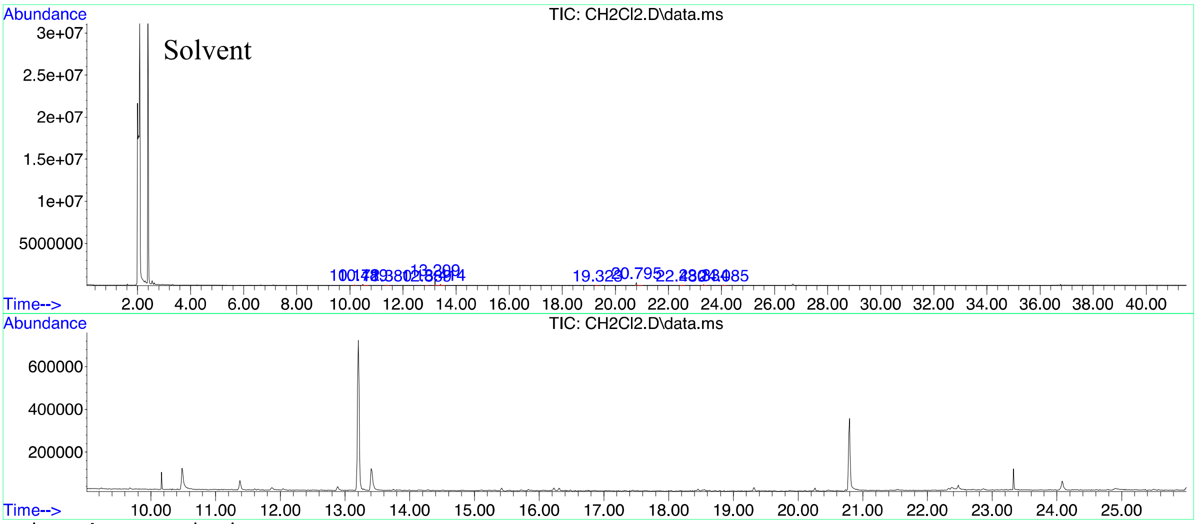


```
Noise characteristics:
  Noise start : 9  min.
  Noise end   : 26 min.
  Number of points: 1675
  Maximum noise: 723442 abundances
  Minimum noise: 16705 abundances
  Peak-to-Peak noise: 706737 abundances
  Root-mean-square noise: 28206.1 abundances

The table below is based on an integrated chromatogram.
Tailing calculated at 10% height.
```

#	RetTime	Area	BslPH	PWidth	Resol	Tailing	PtP S/N	RMS S/N	Plates
1	10.17	570411	65651	0.021	0.00	1.07	0.09	2.33	1263531
2	10.49	2524814	97241	0.038	6.32	2.43	0.14	3.45	425817
3	11.38	890905	42482	0.033	14.90	1.17	0.06	1.51	670268
4	12.89	367735	16209	0.035	26.17	1.05	0.02	0.57	737206
5	13.21	12777297	664673	0.031	5.72	1.06	0.94	23.56	1024488
6	13.41	2540801	99046	0.039	3.47	1.26	0.14	3.51	660434
7	19.32	295196	15049	0.030	101.03	1.49	0.02	0.53	2272962
8	20.79	6248983	324670	0.046	22.78	1.14	0.46	11.51	1128033
9	22.48	635844	20737	0.047	21.32	1.10	0.03	0.74	1255778
10	23.33	851386	82422	0.041	11.47	1.93	0.12	2.92	1828365
11	24.09	1092024	41311	0.039	11.10	1.14	0.06	1.46	2079009

Figure S1. Full chromatogram, extract chromatogram and integrated peaks table.

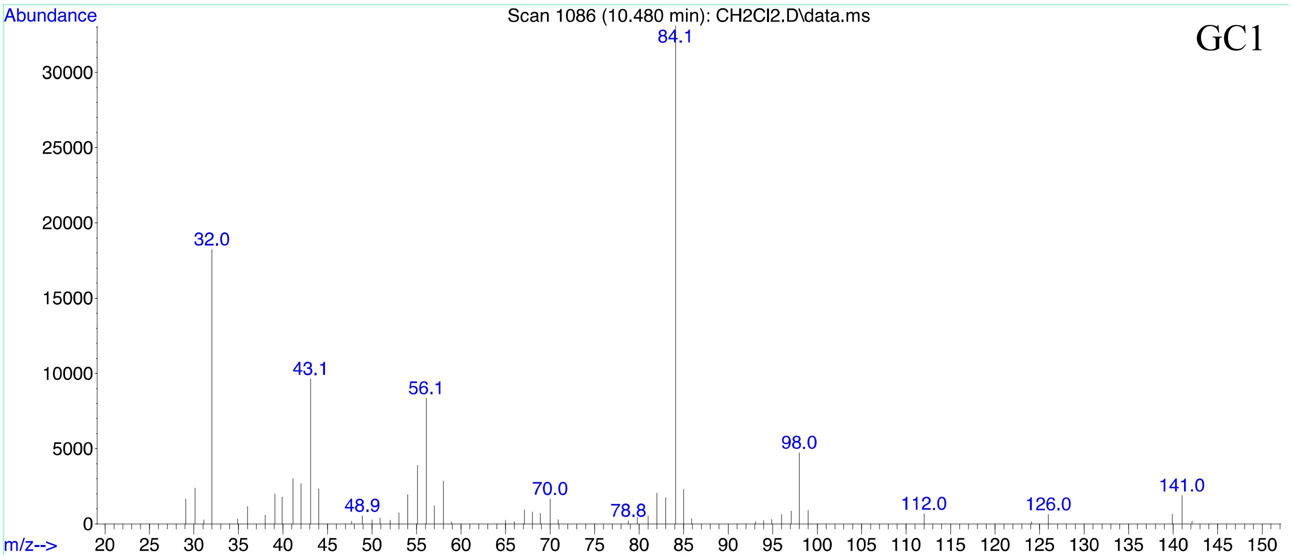

Figure S2. MS spectrum of GC1- Isopelletierine (**1a**).

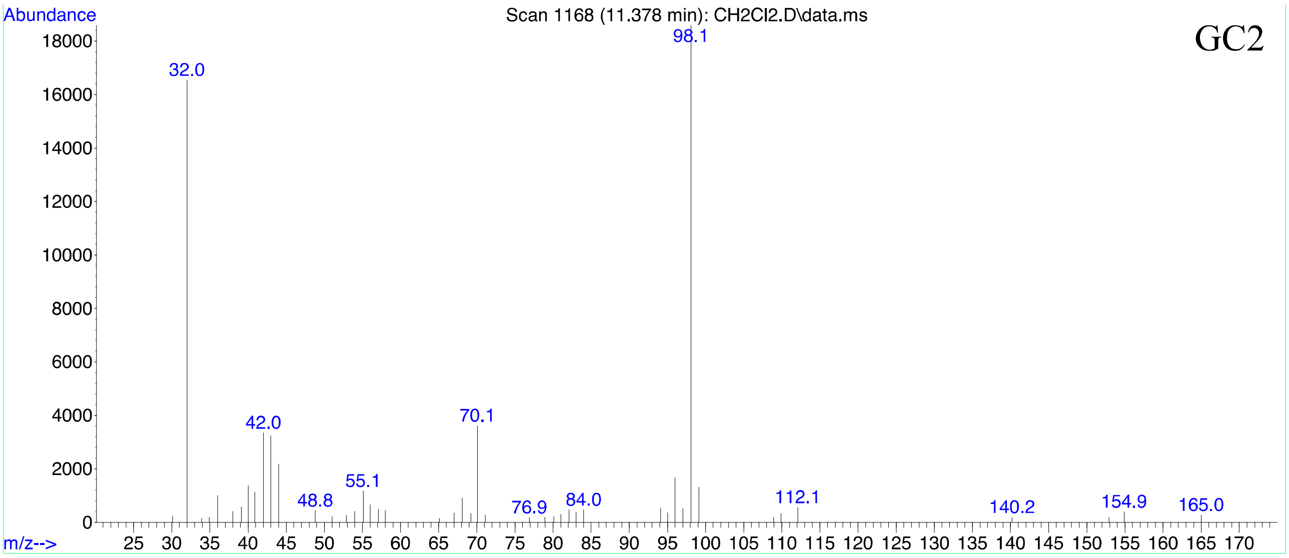

Figure S3. MS spectrum of GC2- Methylpelletierine (**1b**).

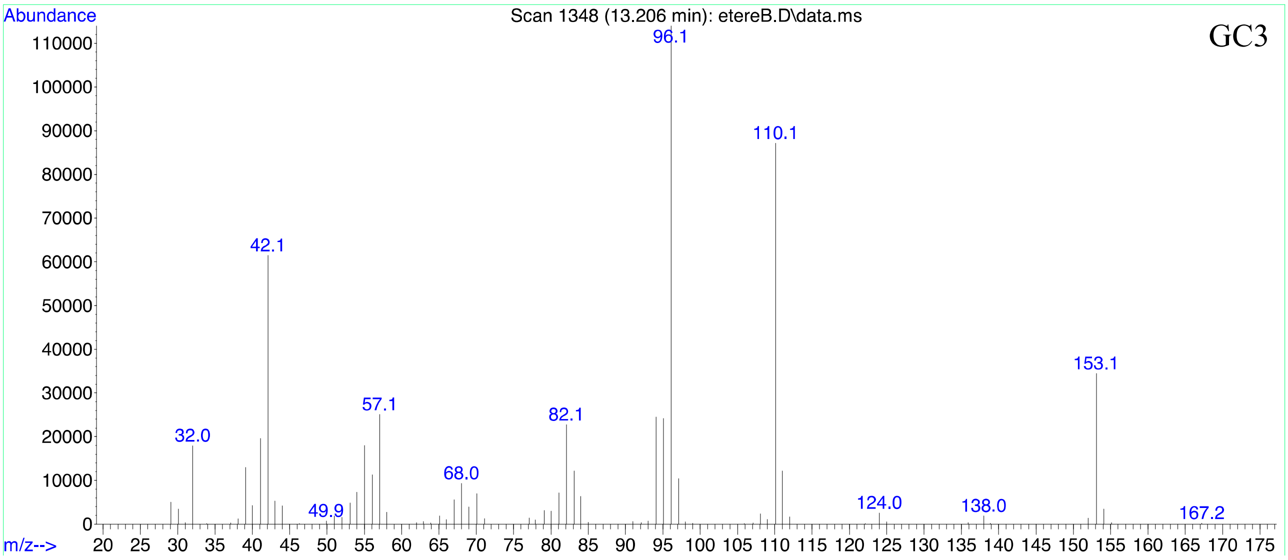

Figure S4. MS spectrum of GC3- Pseudopelletierine (**1c**).

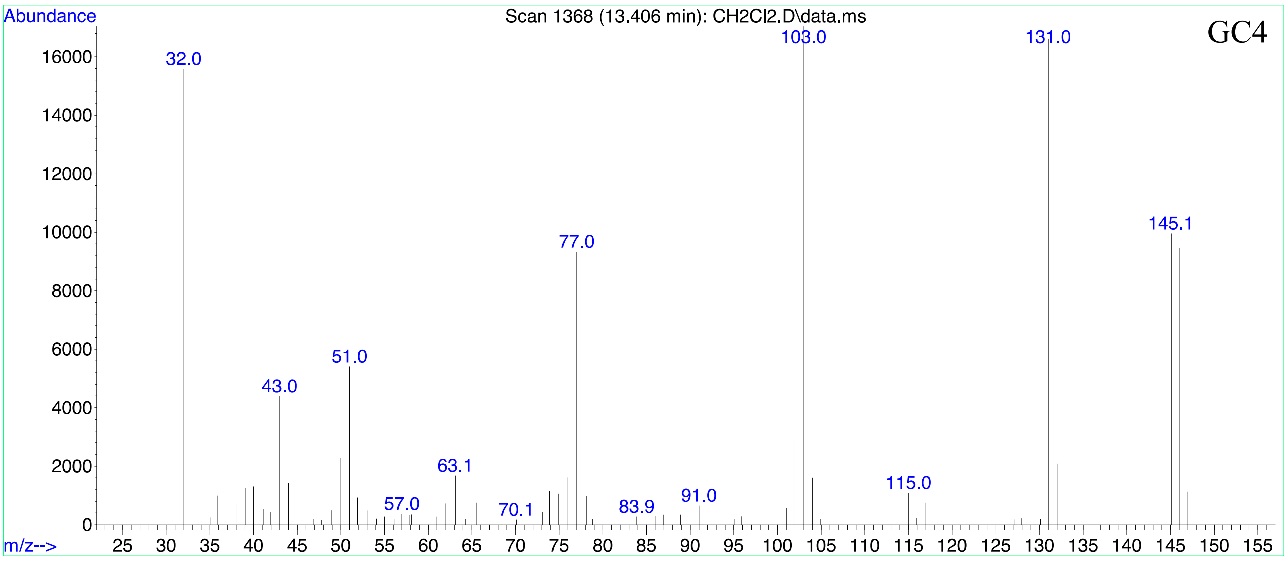

Figure S5. MS spectrum of GC4

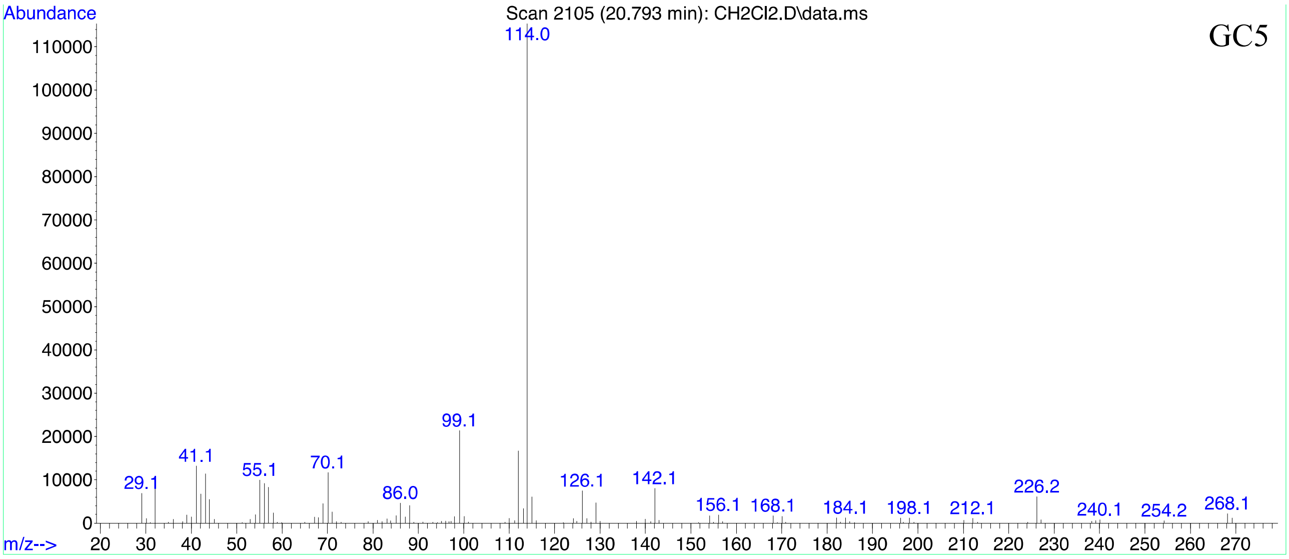

Figure S6. MS spectrum of GC5

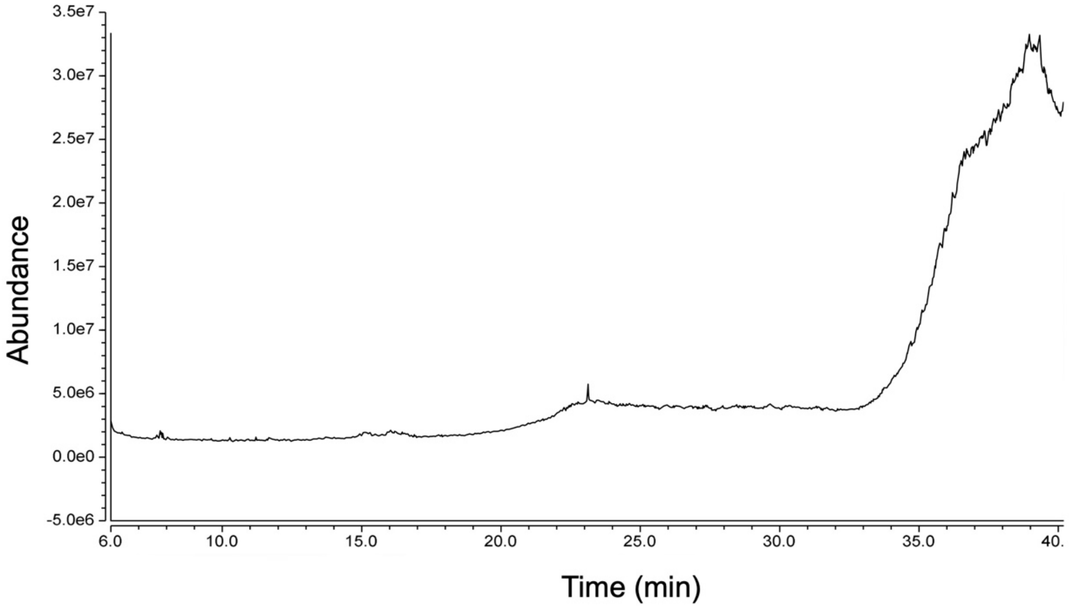

Figure S7. GC-MS analysis of hydroalcoholic (HAE) extract of pomegranate root.

This evidence was confirmed by LC-ESI-HRMS analysis of HAE showing the presence of some phenols, as reported in Figure 3 of the main manuscript, but not the alkaloid compounds.

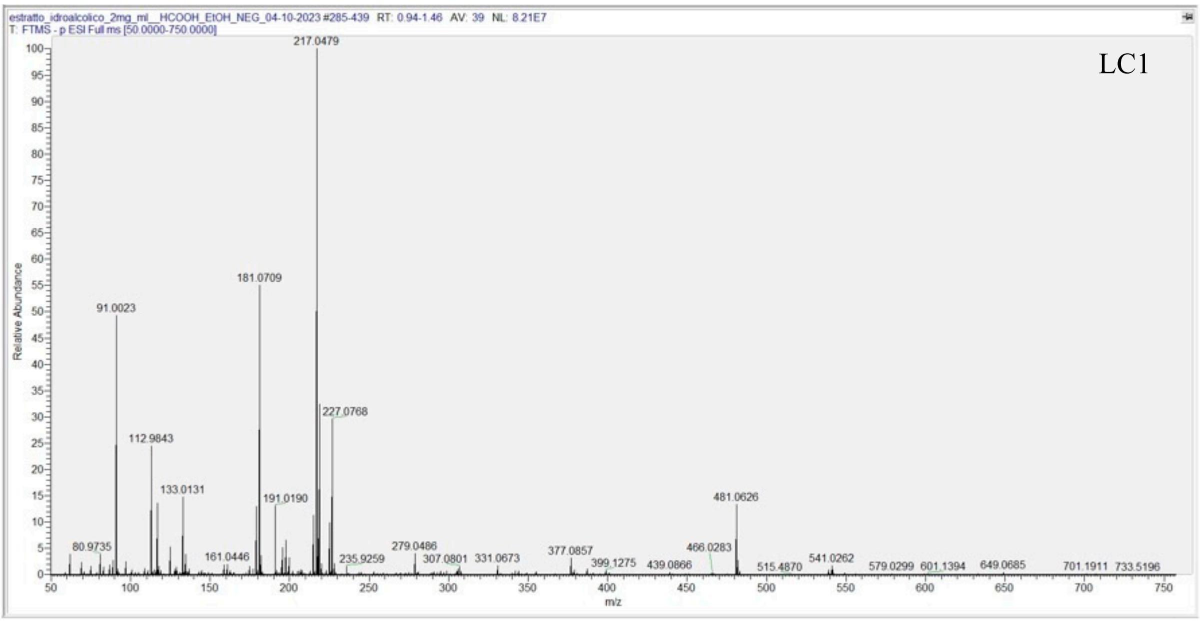

Figure S8. MS spectrum of LC1

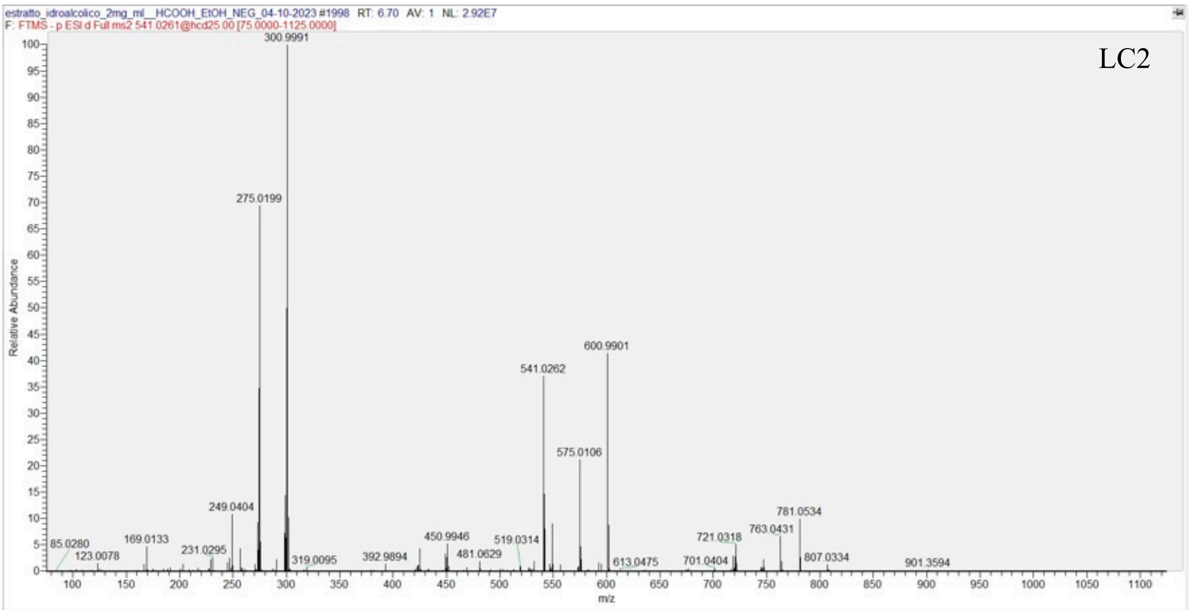

Figure S9. MS spectrum of LC2

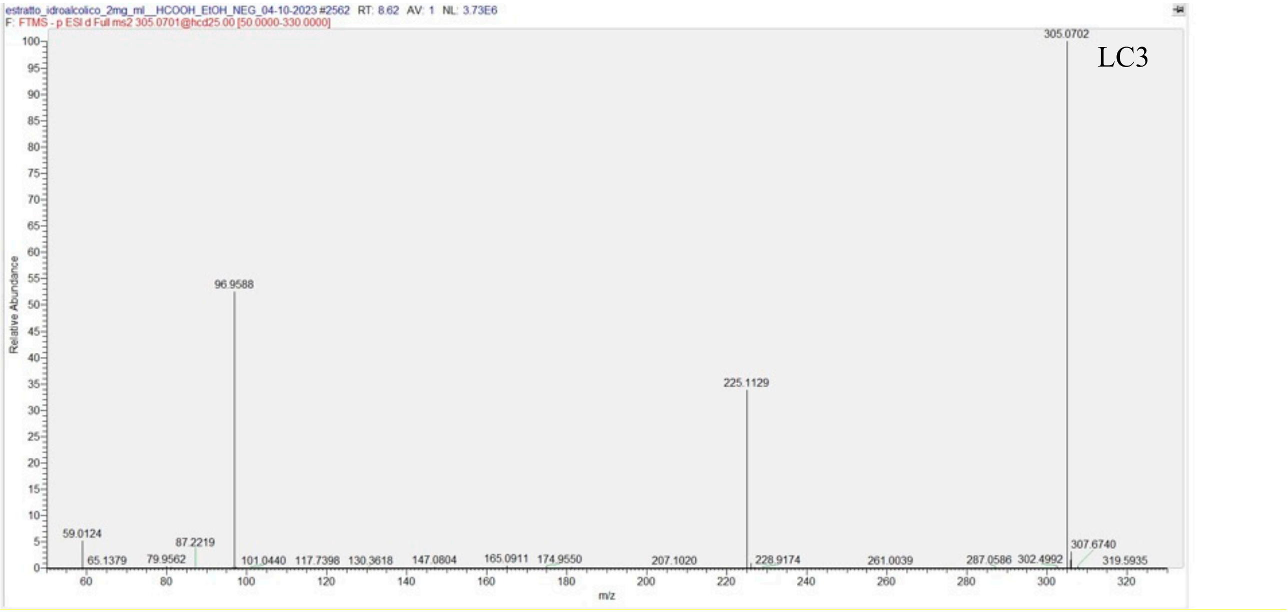

Figure S10. MS spectrum of LC3

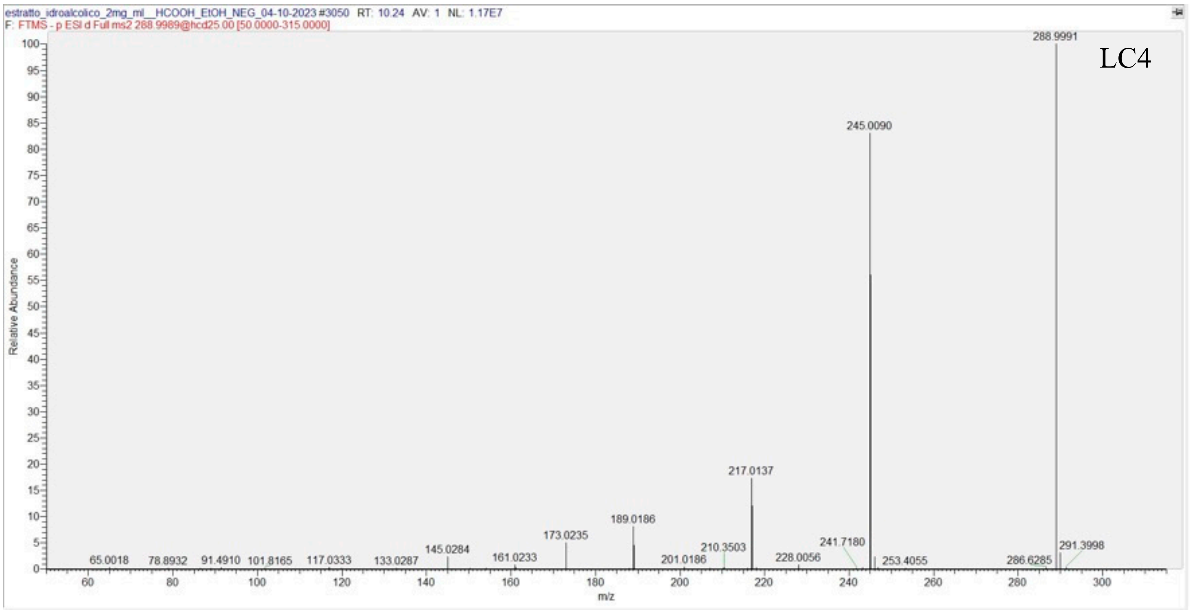

Figure S11. MS spectrum of LC4

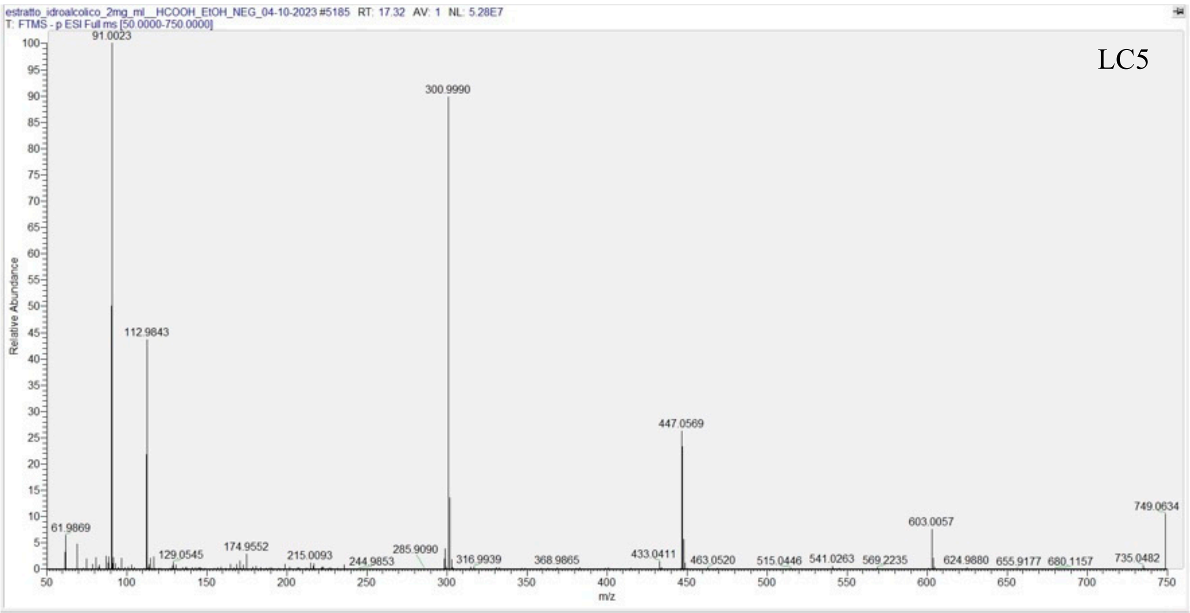

Figure S12. MS spectrum of LC5

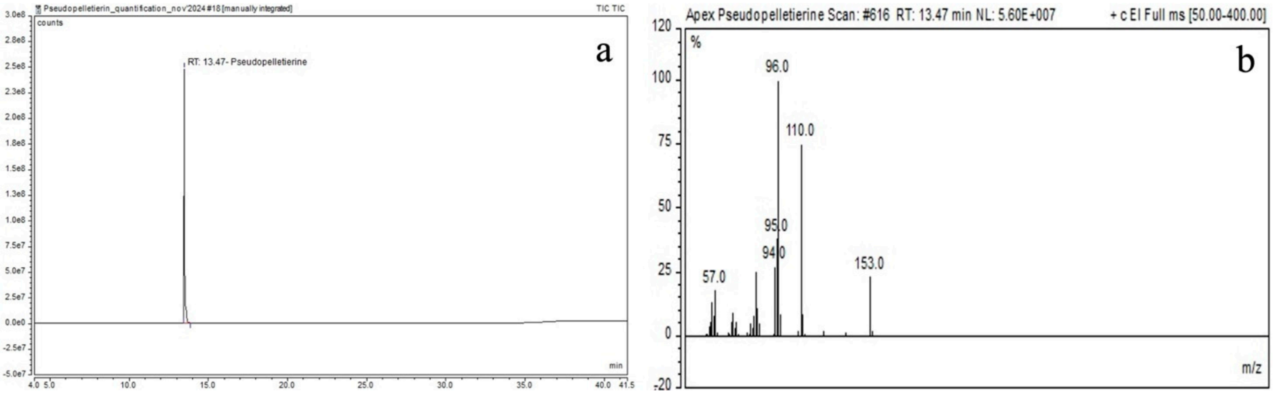

Figure S13. GC-MS chromatogram of isolated pseudopelletierine (1a): (a) Full scan chromatogram (b) EI-MS spectrum

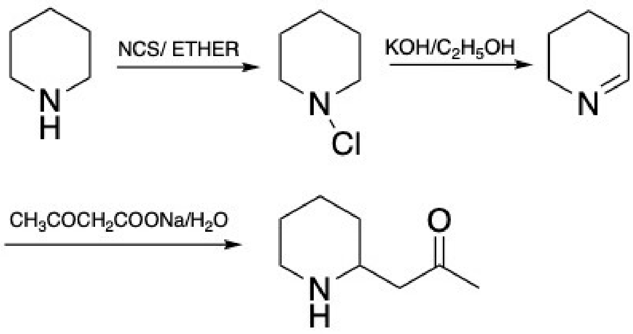

Scheme S1. Synthesis of Isopelletierine.

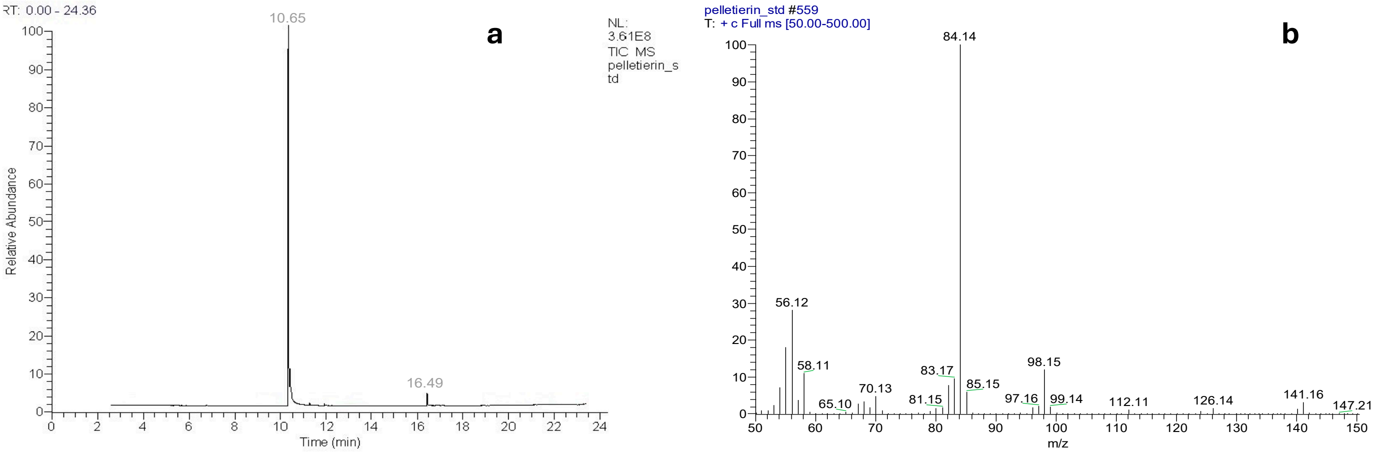

Figure S14. GC-MS chromatogram of synthesized isopelletierine (1a): (a) Full scan chromatogram (b) EI-MS spectrum

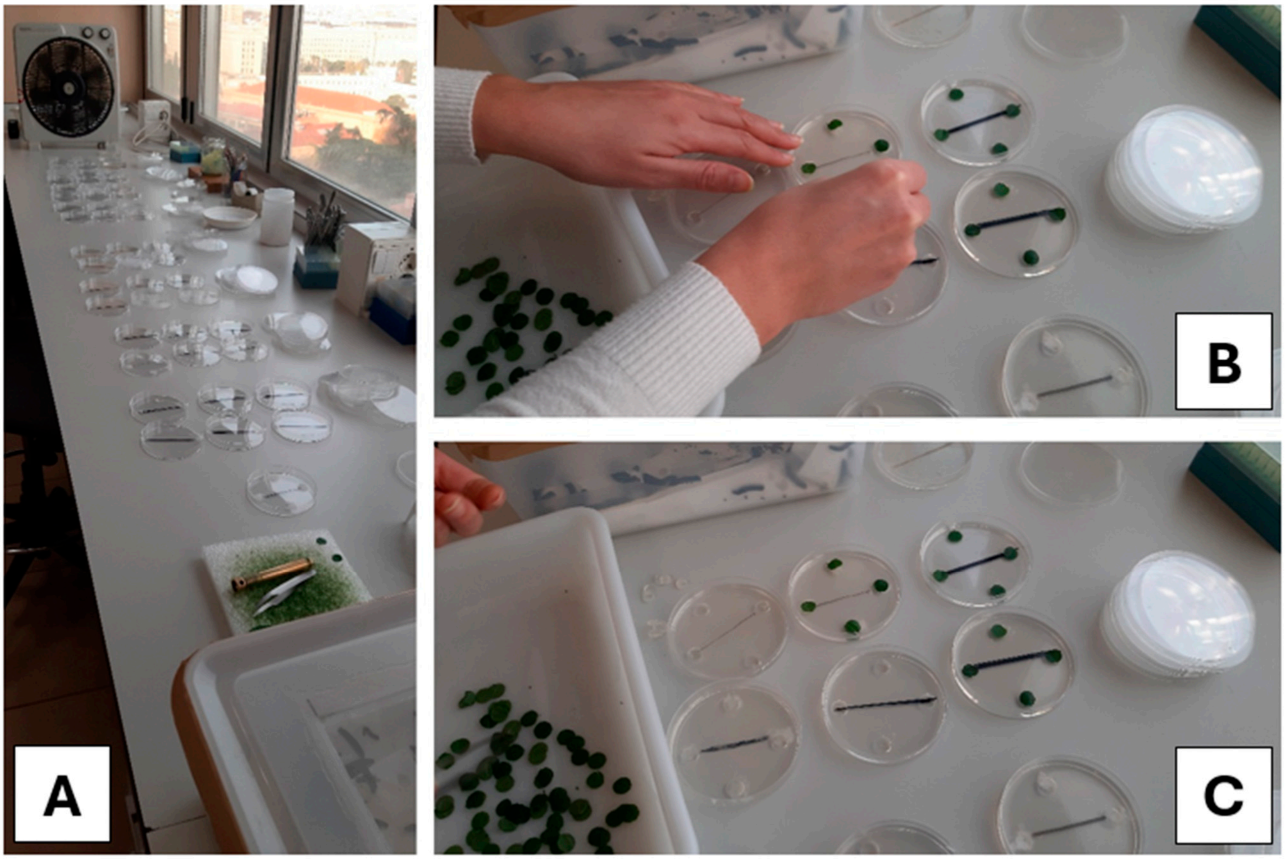

Figure S15. Preparation of anti-food experiment for *Spodoptera littoralis*: ((A) petri dish placement for housing the (B-C) *C. annuum* leaf circular fragments treated with the control (placed on the black vertical mark) and with the samples.

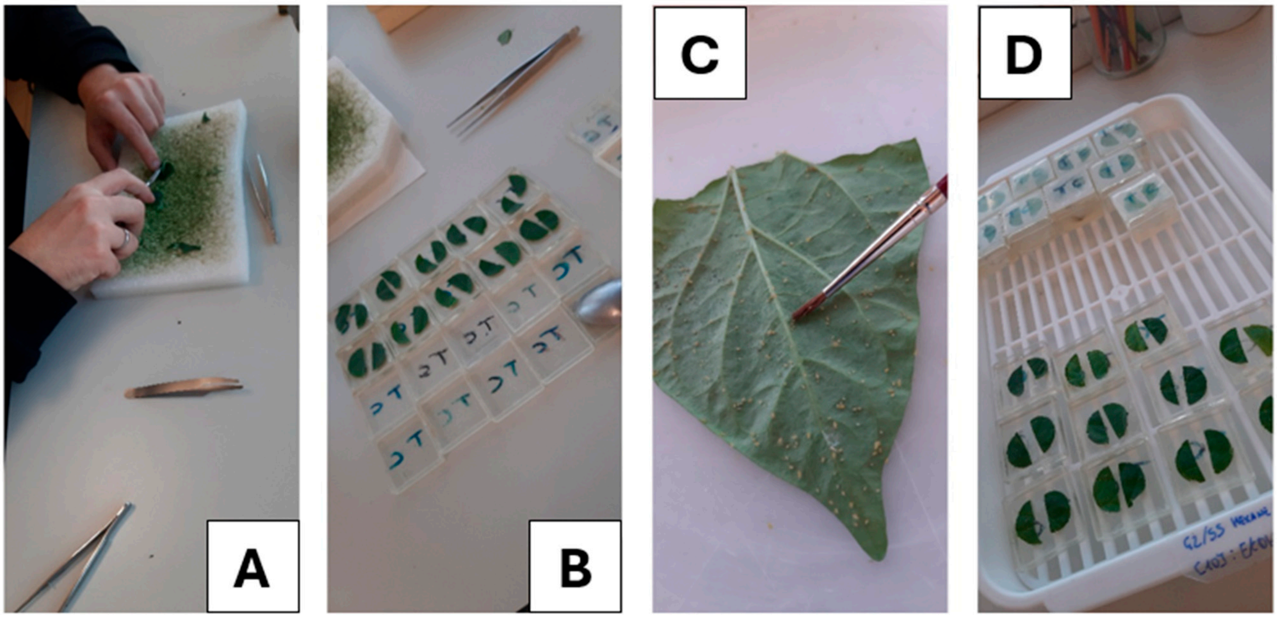

Figure S16. Preparation of anti-stick experiment for *Myzus persicae*: (A-B) ventilated plastic boxes (4 cm^2) placement for housing the *C. annuum* leaf half-circle fragments treated with the control and with the samples; (C-D) *M. persicae* were collected and placed in the boxes with the help of a brush.

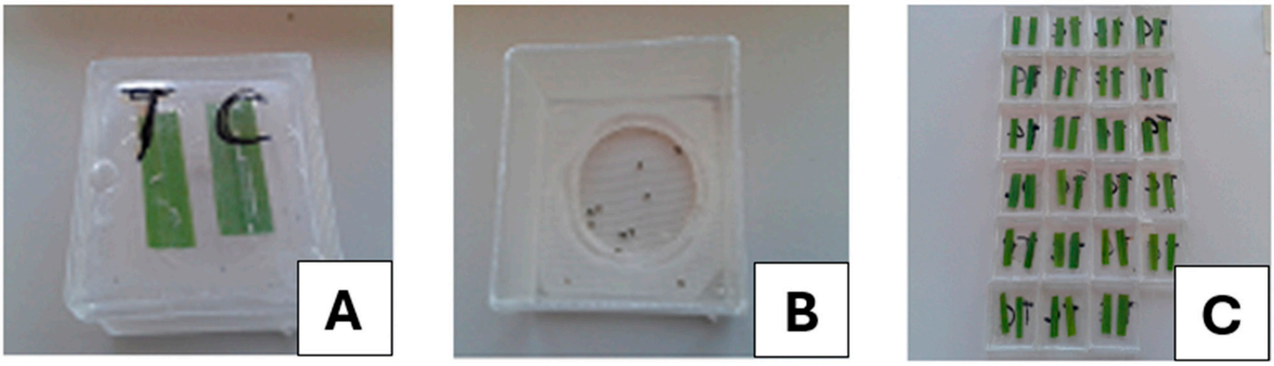

Figure S17. Preparation of anti-stick experiment for *Rhopalosiphum padi*: (A-C) ventilated plastic boxes (4 cm^2) placement for housing the *H. vulgare* leaf fragments treated with the control and with the samples; (B) *M. persicae* were collected and placed in the boxes with the help of a brush.

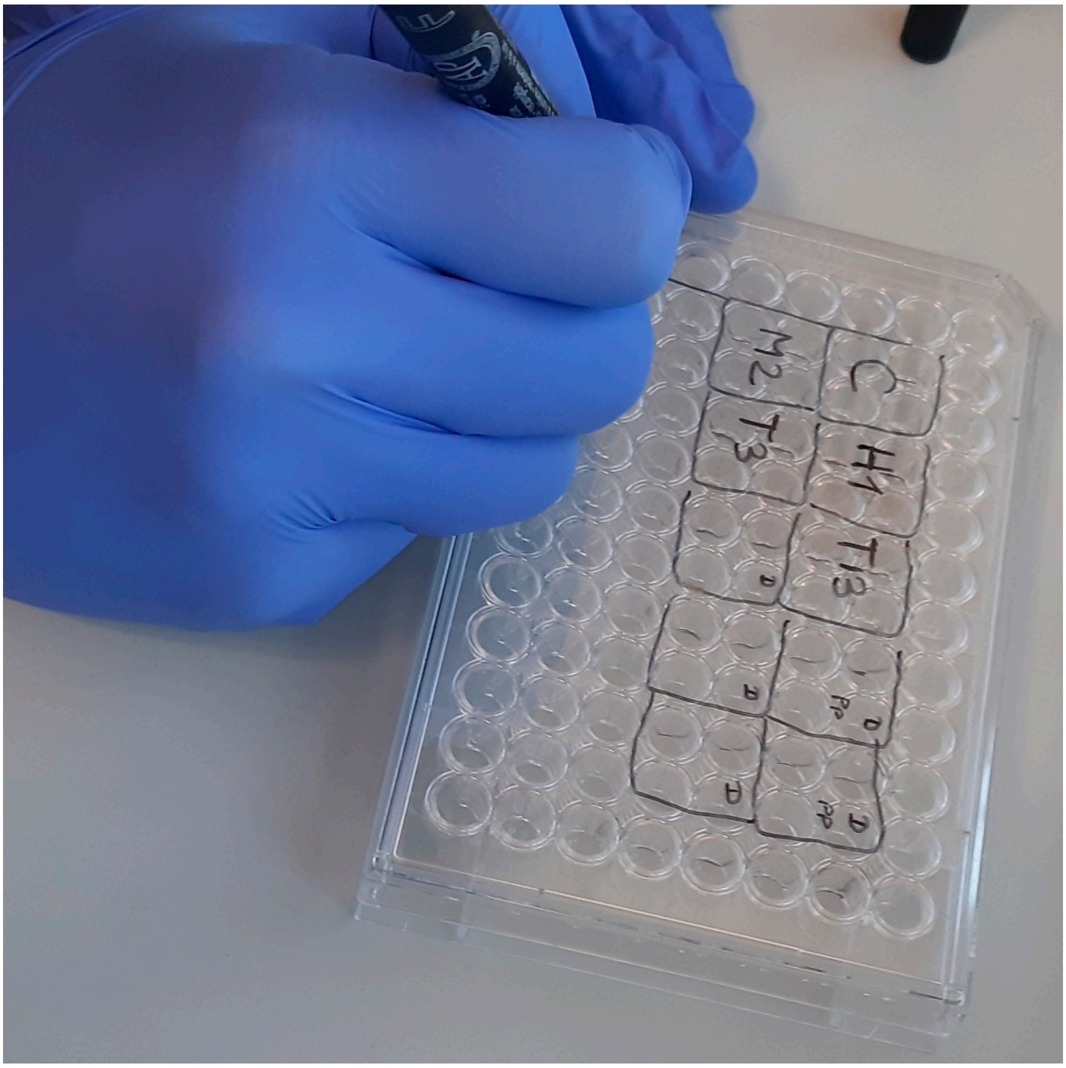

Figure S18. Experiments performed in 96-well plastic plates (U-bottom) with 4 replicates per treatment plus control on *M. javanica*.
